# Personal values in adolescence and sense of coherence in adulthood: A cross‐sectional study based on a retrospective recall

**DOI:** 10.1002/npr2.12111

**Published:** 2020-05-23

**Authors:** Naonori Yasuma, Kazuhiro Watanabe, Daisuke Nishi, Norito Kawakami

**Affiliations:** ^1^ Department of Mental Health Graduate School of Medicine The University of Tokyo Tokyo Japan; ^2^ Department of Community Mental Health and Law National Institute of Mental Health National Center of Neurology and Psychiatry Kodaira Japan

**Keywords:** commitment to values, personal values in adolescence, sense of coherence, value priorities

## Abstract

**Aims:**

The purpose of this study is to retrospectively examine the association between personal values in adolescence and sense of coherence (SOC) in adulthood.

**Methods:**

J‐SHINE data from wave 1 (2010) and wave 3 (2017) were used in this study. We retrospectively measured personal values at age 15 in 2017 in two ways: (a) value priorities developed from Schwartz's theory of basic values; and (b) the commitment to values measured by Personal Values Questionnaire II (PVQ‐II). Multiple regression analysis was used to estimate the association.

**Results:**

Having a value priority of belief, pursuing one's interest, enduring active challenges, cherishing family and friends, and having a commitment to values in adolescence were significantly and positively associated, while avoiding causing trouble was significantly and negatively associated with SOC in adulthood.

**Conclusions:**

Personal values in adolescence may be associated with SOC in adulthood. Research of personal values in adolescence could contribute to the understanding the development of SOC.

## Abstract 2

Personal values in adolescence may be associated with sense of coherence (SOC) in adulthood.

## Graphical abstract

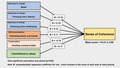

## 1 INTRODUCTION

Sense of coherence (SOC), the core concept of the salutogenic model, was described as the ability to process various stressors by using a variety of resources, the general resistance resources (GRRs).^1^ SOC is composed of three dimensions, comprehensibility, manageability, and meaningfulness.^2^ High SOC is associated with promoting physical and mental health, quality of life and well‐being.^3^ This suggests that health promotion efforts may benefit from strengthening SOC.

Sense of coherence is thought to develop from infancy to early adulthood.^1^ Some researchers stated that SOC should strengthen during adolescence and stabilize toward the end of this developmental period.^4^ Support from family and friends has been positively associated with the development of SOC.^5^, ^6^, ^7^ In addition, gender, socio‐economic status, personality traits, self‐esteem, well‐being, and religiosity could affect positive influence on forming SOC.^8^, ^9^, ^10^, ^11^, ^12^, ^13^ Furthermore, a stable value system could promote the development of high levels of SOC.^14^

Personal values are a broad goal, varying in importance and underlying and guiding attitudes and behaviors.^15^, ^16^ Personal values are formed in adolescence, which may affect long‐term cognitions and behaviors.^15^ A previous study reported that universalistic values which was classified as self‐transcendence were related to the creation of meaningfulness.^17^ Conservative and self‐transcendence values in adolescence have been found to be the most important predictors of SOC in adolescence.^18^ However, the evidence is still limited to one country, Italy, and may not be generalized to other countries with different cultures, such as Asian countries. In addition, personal values in adolescence may have an impact on SOC in adulthood. No studies have investigated the association between personal values in adolescence and SOC in adulthood.

The purpose of this study was to conduct a preliminary and exploratory examination of the association between personal values in adolescence and SOC in adulthood in a large community sample in Japan, by analyzing existing data where the assessment of personal values in adolescence was conducted retrospectively.

## 2 METHODS

### 2.1 Study design, setting, and participants

**Figure 1:**
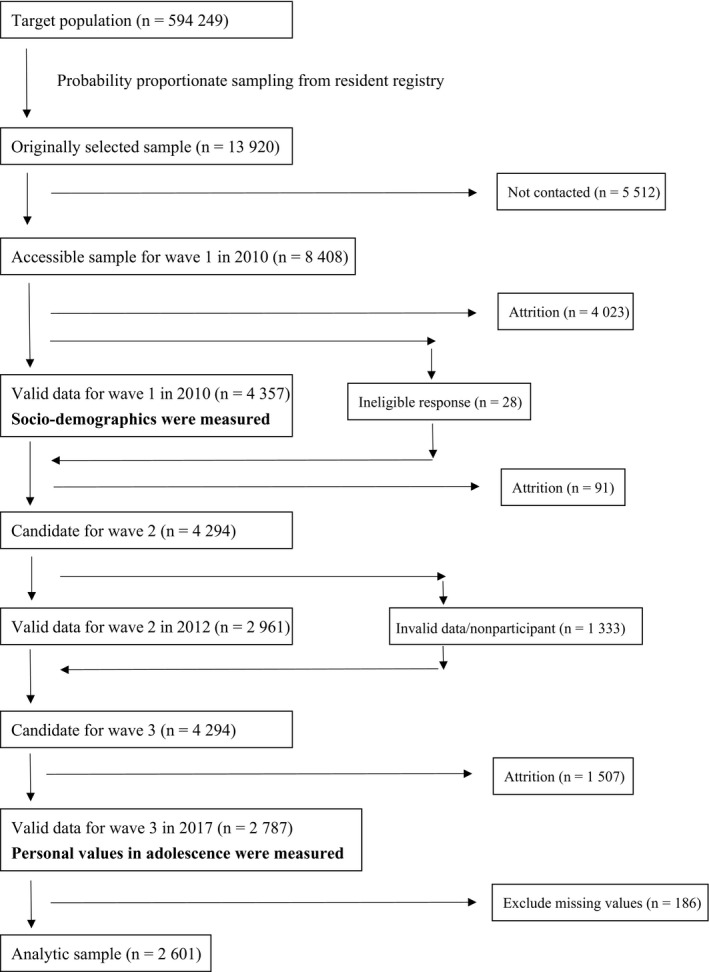
Flowchart of participant recruitment in J‐SHINE

We conducted a cross‐sectional study based on retrospective recall by using wave 1 (2010) and wave 3 (2017) data from the Japanese Study on Stratification, Health, Income, and Neighborhood (J‐SHINE) survey.^19^ Adult residents aged 20‐50 years were randomly selected from four municipalities (two in Tokyo; two in neighboring prefectures) by using systematic sampling methods from a residents’ register. There were no inclusion and exclusion criteria except for age. The participants received an invitation letter, and trained surveyors visited their houses. After the surveyors obtained written informed consent, the participants answered the self‐administered questionnaire with a computer‐aided personal instrument (CAPI). SOC and socio‐demographics were measured in wave 1, and personal values in adolescence were measured in wave 3 (Figure 1). The study protocol was approved by the Research Ethics Committee of the Graduate School of Medicine and the Faculty of Medicine, The University of Tokyo, Japan [No. 630‐73361].

### 2.2 Measures

#### 2.2.1 Personal values in adolescence

Personal values in adolescence were composed of the value priorities and the commitment to values. The value priorities were measured by an 11‐item questionnaire we originally made^20^, ^21^ based on the 57‐item Portrait Values Questionnaire (PVQ‐57).^22^ Eleven value orientations were as follows: avoiding causing trouble, stable lifestyle, belief, pursuing one's interest, enduring active challenges, improving society, cherishing family and friends, positive evaluation, financial success, social influence, graduating from school. These items were rated on a seven‐point Likert scale (1 = not at all, 7 = very important) following the question, “When you were 15‐16 years old, how important did you think the following values were in your life?” Eleven value orientations were also combined into four value dimensions in this study: conservatism (avoiding causing trouble and stable lifestyle), openness to change (belief, pursuing one's interest, enduring active challenges), self‐transcendence (improving society, cherishing family, and friends), and self‐enhancement (positive evaluation, financial success, social influence, and graduating from school). The commitment to values was measured by the Japanese version of the Personal Values Questionnaire II (PVQ‐II).^23^ PVQ‐II consists of eight items (eg, How committed are you to living this value?); the items were rated on a five‐point Likert scale (1 = not at all, 5 = extremely). The reliability and validity of the Japanese version of PVQ‐II have previously been confirmed. In this study, we revised the items to the past tense and instructed the participants to answer the items they considered the most important when they were 15‐16 years old.

#### 2.2.2 Sense of coherence (SOC)

We used the University of Tokyo Health Sociology version of the SOC Scale (SOC‐3‐UTHS) to measure sense of coherence.^24^ It consists of three items (comprehensibility, manageability, and meaningfulness) asked on a seven‐point Likert scale (1 = not at all, 7 = very). The reliability and validity of the Japanese version of SOC‐3‐UTHS have previously been confirmed.

#### 2.2.3 Socio‐demographics

Socio‐demographic information included age, sex, marital status, education, employment, household income, smoking, drinking alcohol, and economic status at 15 years. Education was divided into four groups: junior high school graduates, high school graduates, some college, university graduates, or higher. Employment was divided into three groups, working, on leave, job seeking, housewife, or student. Based on the sum of annual household income, the respondents were divided into five groups: <2.5 million yen, from 2.5 million yen to <5 million yen, from 5 million yen to <7.5 million yen, 7.5 million yen or over, and not known. As for smoking, ex‐smoker was included in smoker. Drinking alcohol was divided into two items: Whether the participants were habitual drinkers (drinking more than three times per week) or not. The economic status at 15 years old was classified as poor, moderate, or good.

### 2.3 Analysis

Multiple regression analysis was used to estimate the association between personal values in adolescence and SOC in adulthood, adjusting for the socio‐demographic variables. A *P‐*value of <.05 was made statistically significant. SPSS (windows version 25) was used for statistical analysis. In these analyses, we did not impute missing responses for the variables.

## 3 RESULTS

### 3.1 Demographic and psychosocial characteristics and SOC in adults

Among the total of 2787 survey respondents, 2669 completed the value priorities and PVQ‐II. Some of the respondents had missing values on the demographic variables of smoking, drinking alcohol, and economic status at age 15 (n = 68) so were excluded from the study. 2601 respondents who did not have missing values were used for analysis. Table 1 shows socio‐demographic variables and SOC in adulthood among the respondents.

**Table 1:** Demographics and psychosocial characteristics of the participants (N = 2601)

Demographic category	N (%)/Mean (SD)
Age	M = 38.09 (SD = 7.03)
Sex (men)	1154 (44.4)
Married (yes)	1956 (75.2)
Education	
Junior high school	79 (3.0)
High school	513 (19.7)
Some college	906 (34.8)
University or higher	1103 (42.4)
Employment	
Working	2011(77.3)
On leave	61 (2.3)
Job seeking or House wife or Students	529 (20.3)
Household Income (per year)	
≦2.5 million yen	176 (6.8)
≦5 million yen	411 (15.8)
≦7.5 million yen	611 (23.5)
>7.5 million yen	826 (31.8)
Not known	577 (22.2)
Current or ever smoking (yes)	1177 (45.3)
Drinking alcohol	
More than 3 times per week	880 (33.8)
<3 times per week	1721 (66.2)
Poor economic status at age 15	489 (18.8)
SOC in adulthood	M = 15.31 (SD = 2.99)

### 3.2 Personal values in adolescence and SOC in adults

Table 2 shows the mean and SD of areas of value priority and the commitment to values. Having a value priority of belief, pursuing one's interest, enduring active challenges, cherishing family and friends, and having a commitment to values in adolescence were significantly and positively associated with SOC in adulthood, adjusting for the socio‐demographic variables. On the other hand, avoiding causing trouble was significantly and negatively associated with SOC in adulthood.

**Table 2:** Personal values in adolescence and SOC in adulthood: multiple regression analysis of data from community residents in Japan (N = 2601)

Areas of four value dimensions	Areas of value priority: (1‐7)	Mean (SD)	SOC in adulthood^a^		
			B	SE	*P*
Conservatism	Avoiding causing trouble	5.58 (1.36)	‐0.10	0.05	.048*
	Stable lifestyle	4.86 (1.43)	‐0.001	0.05	.98
Openness to change	Belief	4.83 (1.41)	0.18	0.05	<.01**
	Pursuing one's interest	5.13 (1.40)	0.10	0.05	.047*
	Enduring active challenging	4.50 (1.43)	0.12	0.05	.03*
Self‐transcendence	Improving society	3.77 (1.43)	0.02	0.05	.73
	Cherishing family and friends	5.54 (1.25)	0.11	0.05	.04*
Self‐enhancement	Positive evaluation	4.90 (1.40)	‐0.01	0.05	.82
	Financial success	4.22 (1.53)	‐0.10	0.05	.06
	Social influence	3.30 (1.41)	0.01	0.05	.83
	Graduating from school	4.26 (1.67)	0.02	0.04	.62
Commitment to values (8‐40)		26.35 (4.76)	0.09	0.01	<.01**
Intercept			9.23	0.69	<.01**

^a^ Unstandardized regression coefficient (B) for one‐point increase in the score of each area of value priority is shown, adjusting for socio‐demographic variables (age, sex, marital status, education, employment, household income, smoking, drinking alcohol, and economic status at 15 years).

* *P* < .05,

** *P* < .01.

## 4 DISCUSSION

To our knowledge, this is the first study to investigate the association between personal values in adolescence and SOC in adulthood in a country outside of Europe. Having a value priority of belief, pursuing one's interest, enduring active challenges, cherishing family and friends, and having a commitment to values in adolescence were significantly and positively associated with SOC in adulthood, while avoiding causing troubles was significantly and negatively associated.

The previous study showed that having conservative and self‐transcendence values were positively associated with SOC in adolescence.^18^ Our results were inconsistent with the previous study.^18^ The possible reasons for this inconsistency were as follows. First, the timing of SOC measurement differed between the two studies. The values influencing SOC formation may have been different between adolescence and adulthood. Second, religious differences that may provide a coherent global framework could affect the inconsistency.^25^ The previous study was conducted in Italy while our study was performed in Japan. As many Italians believe in Catholicism, living experience with stable rules and norms with Catholics' conservative values may have affected the formation of SOC.^26^ On the other hand, in Japan, which was reported as less religious than other countries, having a successful coping experience under moderate stress with openness to change values might have been more significant for creating SOC.^27^ Third, the measurement of value priorities in adolescence varied between the two studies. The reliability and validity of our original questionnaire had not been fully confirmed, which could have caused random error.^20^, ^21^ However, our study found that having a value priority of belief, pursuing one's interest, enduring active challenges, cherishing family and friends, and having a commitment to values in adolescence were associated with SOC in adulthood. The possible mechanism of this finding could be explained by the Antonovsky's theory.^1^ Antonovsky stated that the development of SOC required three types of experience: living experience with stable rules and norms, successful coping experience under moderate stress, and participation in important decision‐making.^1^ Having a value priority of belief, pursuing one's interest, enduring active challenges, cherishing family and friends, and having a commitment to values in adolescence might offer such experiences.

This study has three strengths. First, the topic of personal values in adolescence had not been fully researched. Second, as a practical implication, moral education about having a value priority of belief, pursuing one's interest, enduring active challenges, cherishing family and friends, and having a commitment to values in adolescence could have positive effects on future SOC. Third, most SOC studies were conducted in the West, while this study was performed in Asia. However, there were many limitations. First, the considerably large loss of follow‐up in the study population and some unbalance socio‐demographic characters such as fewer men (44.4%) and a very high rate of married participants (75.2%) could have caused selection bias. Second, recall bias may have occurred because the participants had to remember the values that were important to them when they were 15 years old. Third, childhood adversity should be taken into account as a confounding factor because it can affect SOC and presumably would be associated with personal values in adolescence. Fourth, reliability and validity of the measurement of value priorities in adolescence have not been fully evaluated, so random error may have occurred. Fifth, as this study was cross‐sectional, a cohort study should be conducted to clarify the causality of this association.

## 5 CONCLUSIONS

This retrospective study suggested a possible association between the personal values in adolescence and SOC in adulthood. Personal values in adolescence related to self‐transcendence and openness to change could make a significant contribution to understanding and having positive effects on SOC in adulthood.

## CONFLICT OF INTERESTS

None.

## DATA REPOSITORY

The data that support the findings of this study are proprietary data of the J‐SHINE study and therefore are not publicly available. Restrictions apply to the availability of these data, which were used under license for this study. However, the data might be available from the Data Management Committee of the J‐SHINE study by contacting the Chair, Prof Hideki Hasimoto (hidehashimoto‐circ@umin.ac.jp).

## APPROVAL OF THE RESEARCH PROTOCOL BY AN INSTITUTIONAL REVIEWER BOARD

The study was approved by the University of Tokyo ethics committee.

## INFORMED CONSENT

Informed consent was obtained from all subjects.

## AUTHOR CONTRIBUTIONS

NY wrote the manuscript and KW, DN, and NK critically revised the manuscript.
